# Tetany Exacerbating Heart Failure: A Case Report

**DOI:** 10.7759/cureus.12467

**Published:** 2021-01-04

**Authors:** Junya Tanabe, Shohei Fukunaga, Akihiro Endo, Takafumi Ito, Kazuaki Tanabe

**Affiliations:** 1 Cardiology, Shimane University Faculty of Medicine, Izumo, JPN; 2 Nephrology, Shimane University Faculty of Medicine, Izumo, JPN

**Keywords:** hypocalcemia, renal dysfunction, ischemic cardiomyopathy

## Abstract

Tetany is characterized by numbness and stiffness in the hands and feet caused by hypocalcemia, hypomagnesemia, and hyperventilatory alkalosis, primarily at peripheral neuromuscular junctions. Although hypocalcemia is common in critically ill patients, its diagnosis of hypocalcemia is complicated and sometimes overlooked. We encountered an 82-year-old woman with tetany that exacerbated heart failure. Pain and respiratory failure due to tetany are conditions that can lead to exacerbation of heart failure. Chronic renal failure is frequently associated with chronic heart failure, and regular follow-up of calcium, phosphorus, and magnesium levels is necessary for such patients.

## Introduction

Tetany is characterized by numbness and stiffness in the hands and feet caused by hypocalcemia, hypomagnesemia, and hyperventilatory alkalosis, primarily at peripheral neuromuscular junctions. Often, the etiology is not a single cause but rather a combination of electrolyte abnormalities. The clinical presentation of hypocalcemia ranges from an asymptomatic biochemical abnormality to a life-threatening disorder. Moderate to severe hypocalcemia is associated with increased paresthesia and neuromuscular hypersensitivity in the fingers, toes, and around the lips. Severe hypocalcemia can cause painful muscle spasms of the extremities as well as asthma-like symptoms owing to bronchospasm and is associated with a risk of asphyxiation owing to laryngospasm [1, 2]. Although hypocalcemia is common in critically ill patients, its diagnosis of hypocalcemia is complicated and sometimes overlooked [3]. Low serum levels of total and ionized calcium directly alter myocyte function and hypocalcemia has been shown to contribute to cardiac dysfunction [4]. We report a case of tetany that exacerbated heart failure.

## Case presentation

The patient was an 82-year-old woman who had a 3-year history of leg cramps and first had chest tightness for two years. She had been treated for ischemic cardiomyopathy with triple vessel disease at another hospital, but had been hospitalized for heart failure 12 times in the last two years. Echocardiography that was performed in the hospital reportedly revealed a left ventricular ejection fraction (LVEF) of 30%. The patient had a history of left thyroidectomy for thyroid cancer at the age of 67 years with no subsequent recurrence. Comorbidities included angina pectoris, chronic heart failure, hypertension, type 2 diabetes mellitus, chronic renal failure, and gastric ulcer (gastrointestinal bleeding with antithrombotic drugs). Her usual medications were tolvaptan 7.5 mg, azosemide 60 mg, furosemide 60 mg, rabeprazole 10 mg, amlodipine 5 mg, bisoprolol 0.625 mg, amiodarone 50 mg, levothyroxine sodium 50 μg, cremedin 6 g, and sodium ferrous citrate 100 mg. Her ability to perform activities of daily living was good, and there was no history of alcohol drinking, smoking, or allergy. She noticed shortness of breath and chest discomfort when she was about to go to the restroom in the morning, and was transported by ambulance to the emergency department of our hospital.

**Figure 1:**
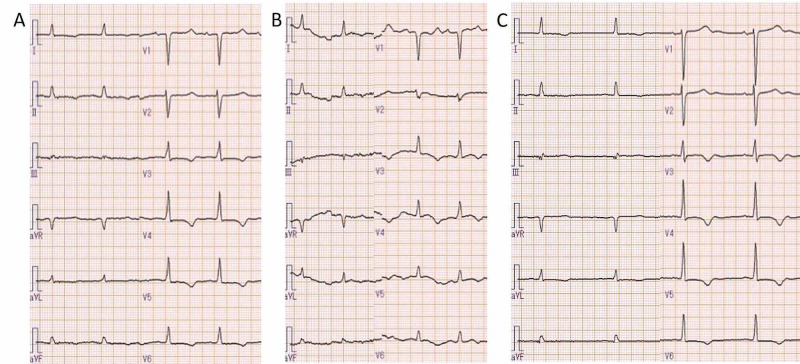
On admission, a 12-lead ECG (A) showed a sinus rhythm at 71 beats/min, no ST elevation, and a negative T wave in V3-6. Twelve hours after admission, the patient complained of sudden chest pain, and the ECG (B) showed no significant difference from that obtained at the time of admission. At the time of discharge, the QTc decreased from 556 to 491 ms (C).

Physical findings on admission were: height, 153 cm; weight, 53 kg; body mass index, 22.6 kg/m^2^; body temperature, 36.9℃; blood pressure, 155/94 mmHg; heart rate, 83/min and regular; respiratory rate, 22/min; and SpO_2,_ 98% (O_2_, 6 L). The palpebral conjunctiva was not pale, and jugular venous distention was observed. There were coarse crackles in both lung fields and no murmurs. There was no peripheral coldness in the limbs and lower leg edema was observed. A 12-lead electrocardiography (ECG) showed sinus rhythm at 71 beats/min, no ST elevation, and a negative T wave in leads V3-6 (Figure 1A). The corrected QT interval (QTc) was prolonged by 556 ms.

**Figure 2:**
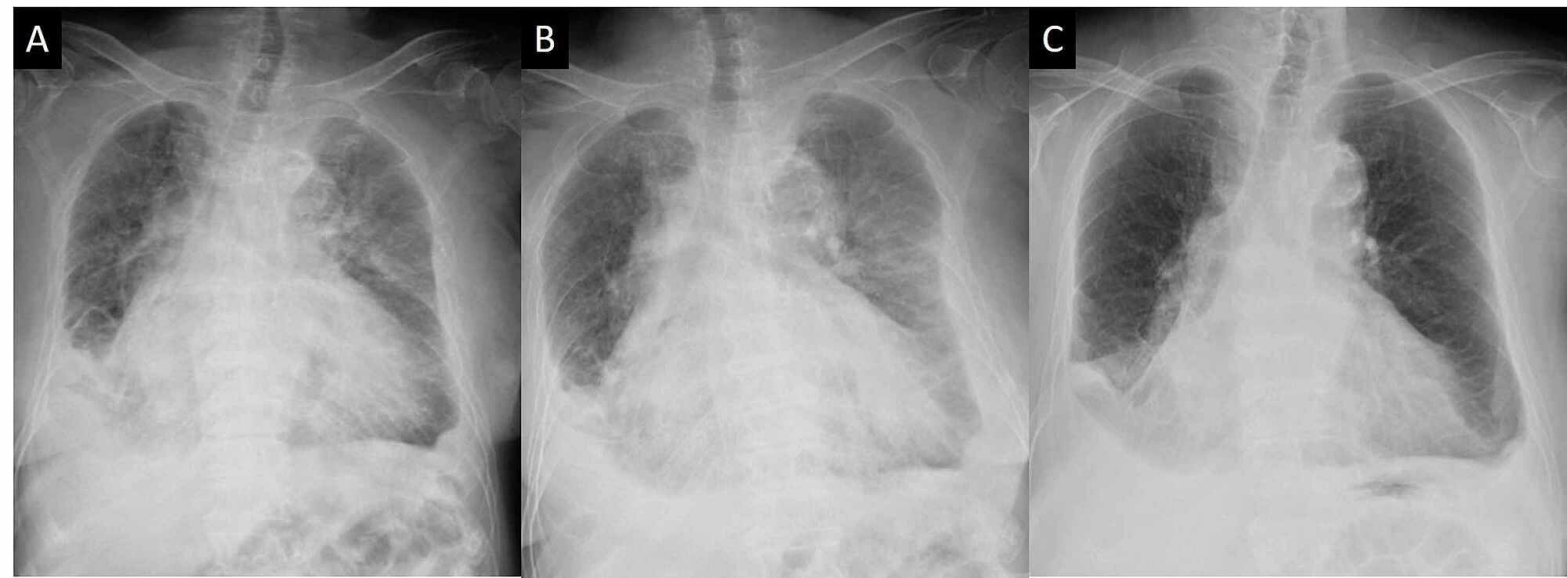
Chest radiography (A): Chest radiography on admission. The cardiothoracic ratio was 70%, indicating cardiomegaly, and pulmonary congestion and bilateral pleural effusion could be noted. (B): Twelve hours after admission, the patient complained of sudden onset chest pain and the chest radiography showed worsening pulmonary congestion. (C): Chest radiography obtained at the time of discharge.

Chest radiography showed a cardiothoracic ratio of 70%, indicating cardiomegaly, pulmonary congestion, and bilateral pleural effusion (Figure 2A).

Echocardiography showed akinesis mainly at the apex and diffuse hypokinesis at the base, showing systolic dysfunction with LVEF of 15%. The blood tests revealed decreased renal function with a blood urea nitrogen (BUN) level of 88 mg/dL, creatinine level of 3.26 mg/dL, and estimated glomerular filtration rate (eGFR) of 11.1 mL/min/BSA.The serum albumin level was 3.3 g/dL and serum potassium level was 4.5 mmol/L. Creatine kinase (CK) was slightly elevated at 191 U/L, but the creatine kinase-myocardial band (2.8 ng/mL) and troponin I (0.02 ng/mL) levels were not elevated. The level of brain natriuretic peptide (BNP) was 802.7 pg/mL. A diagnosis of acute exacerbation of chronic heart failure was made, and treatment with oxygen and diuretics (furosemide 20mg) was started. The patient responded well and urine volumes in the initial three hours were 400 mL; however, 12 hours after admission, she experienced sudden onset chest pain and pain radiating to the neck. Consciousness at the time of the attack was clear, systolic blood pressure was 120 mmHg, heart rate was 80/min with sinus rhythm, and SpO_2_ was 89% (O_2_ 10 L). The pain extended to the extremities. The 12-lead ECG at the time of the attack was not significantly different from that at the time of admission (Figure 1B). Chest radiography showed worsening pulmonary congestion (Figure 2B). Echocardiography and blood sampling showed no marked change from that on admission, no increase in cardiac enzymes, and no findings strongly suggestive of acute coronary syndrome. When respiratory management by noninvasive positive pressure ventilation was continued, symptoms improved after approximately one hour. Although serum calcium and magnesium levels were not measured at the time of admission, additional examinations revealed hypocalcemia with a calcium level of 4.9 mg/dL and hypomagnesemia with a magnesium level of 1.7 mg/dL. The phosphorus level was 6.3 mg/dL. It was considered that the symptoms were due to a tetanic seizure. The cause of hypocalcemia was thought to be chronic kidney disease (CKD) because parathyroid function, as reflected by intact parathyroid hormone (PTH), was preserved (122.7 pg/mL) despite prior operation on the thyroid gland. Since the level of consciousness and symptoms did not deteriorate, calcium correction was started by oral administration while magnesium was corrected by drip infusion. The patient rapidly responded to intravenous infusion of magnesium and oral administration of calcium and vitamin D preparations. Serum calcium normalized on day 6 of hospitalization and the corrected calcium level was 9.1 mg/dL at the time of discharge. The QTc on ECG decreased from 556 to 491 ms (Figure 1C). The electrolyte abnormality improved without subsequent recurrence of tetany. The LVEF by echocardiography improved from 15% to 27% at the time of discharge. Although coronary angiography was not performed because of renal dysfunction, the LV contraction failure may have been affected by hypocalcemia. Her heart failure was well controlled with tolvaptan 15 mg, azosemide 90 mg, furosemide 20 mg and and bisoprolol 0.625 mg (Figure 2C), and she left hospital on the 13th day of hospitalization.

## Discussion

Approximately 40% of calcium is bound to albumin and is tightly regulated by PTH and vitamin D. Hypomagnesemia is a common electrolyte disturbance that occurs in up to 12% of hospitalized patients and up to 60 to 65% of patients in the intensive-care unit [5, 6]. The causes of hypocalcemia vary, with impaired production of PTH and vitamin D being the most common. Hypoparathyroidism in adults generally results from accidental damage to all four glands as a result of thyroid or parathyroid surgery. Vitamin D deficiency, particularly impaired production of 1, 25-dihydroxyvitamin D, occurs primarily secondary to renal failure [7]. In this case, although the patient had a history of left thyroidectomy, blood tests showed that the parathyroid gland function was preserved along with uncontrolled CKD. Hypocalcemia was considered to be caused by a bone mineral metabolism abnormality associated with CKD, including 1, 25-dihydroxyvitamin D production failure. In addition, loop diuretics inhibits Na^+^-K^+^-2Cl^-^ cotranspoter, thereby decreasing luminal positive charges and reducing calcium and magnesium reabsorption [8]. The pain caused by tetany and difficulty in breathing caused by heart failure were thought to lead to a vicious cycle of worsening tetany and heart failure with pain in this patient.

Low serum levels of total and ionized calcium directly alter myocyte function [9-11]. Hypocalcemia prolongs phase 2 of the action potential duration and the QT interval. Severe hypocalcemia can impair cardiac contractility and leads to diffuse musculoskeletal syndrome comprising tetany and rhabdomyolysis. When hypocalcemia is severe, patients may experience bronchospasm or laryngospasm that mimics asthma. Localized or generalized seizures may also occur. These conditions are resistant to conventional treatment for heart failure. In this case, the level of serum calcium was not measured before admission, hypocalcemia has been shown to contribute to cardiac dysfunction and hypotension. Additionally, calcium administration improves blood pressure and ventricular function in critically ill patients with hypocalcemia [12].

Rapid calcium correction via intravenous injection is desirable when symptoms of severe hypocalcemia are noted [13, 14]. Acute hypocalcemia can result in severe symptoms requiring hospitalization, whereas patients who gradually develop hypocalcemia are more likely to be asymptomatic. Failure to normalize severe hypocalcemia is associated with increased mortality [3, 15]. Patients with chronic heart failure have a high rate of chronic renal failure and the potential to develop abnormalities in the levels of electrolytes such as calcium, magnesium, and phosphorus. Hypocalcemia is a rare cause of reversible congestive heart failure that should be part of the differential diagnosis in any patient presenting with heart failure who does not respond to therapy for heart failure [16-18].

## Conclusions

We encountered a case of tetany that exacerbated heart failure. Pain and respiratory failure due to tetany are conditions that can lead to exacerbation of heart failure. Chronic renal failure is frequently associated with chronic heart failure, and regular follow-up of calcium, phosphorus, and magnesium levels is necessary for such patients.

## References

[1] Fong J, Khan A (2012). Hypocalcemia: updates in diagnosis and management for primary care. Can Fam Physician.

[2] Bove-Fenderson E, Mannstadt M (2018). Hypocalcemic disorders. Best Pract Res Clin Endocrinol Metab.

[3] Kelly A, Levine MA (2013). Hypocalcemia in the critically ill patient. J Intensive Care Med.

[4] Jankowski S, Vincent JL (1995). Calcium administration for cardiovascular support in critically ill patients: when is it indicated?. J Intensive Care Med.

[5] Agus ZS (1999). Hypomagnesemia. J Am Soc Nephrol.

[6] Uehara A, Kita Y, Sumi H, Shibagaki Y (2019). Proton-pump inhibitor-induced severe hypomagnesemia and hypocalcemia are clinically masked by thiazide diuretic. Intern Med.

[7] Hannan FM, Thakker RV (2013). Investigating hypocalcemia. BMJ.

[8] Lee CT, Chen HC, Lai LW, Yong KC, Lien YH (2007). Effects of furosemide on renal calcium handling. Am J Physiol Renal Physiol.

[9] Bogin E, Massry SG, Harary I (1981). Effect of parathyroid hormone on rat heart cells. J Clin Invest.

[10] Rampe D, Lacerda AE, Dage RC, Brown AM (1991). Parathyroid hormone: an endogenous modulator of cardiac calcium channels. Am J Physiol.

[11] Nakano S, Masuda K, Asanuma T, Nakatani S (2016). The effect of chronic renal failure on cardiac function: an experimental study with a rat model. J Echocardiogr.

[12] Sauter TC, Lindner G, Ahmad SS, Leichtle AB, Fiedler GM, Exadaktylos AK, Haider DG (2015). Calcium disorders in the emergency department: independent risk factors for mortality. PLoS One.

[13] Johnson MM, Patel S, Williams J (2019). Don’t take it ‘Lytely’: a case of acute tetany. Cureus.

[14] Michels TC, Kelly KM (2013). Parathyroid disorders. Am Fam Physician.

[15] Steele T, Kolamunnage-Dona R, Downey C, Toh CH, Welters I (2013). Assessment and clinical course of hypocalcemia in critical illness. Critical Care.

[16] Kazmi AS, Wall BM (2007). Reversible congestive heart failure related to profound hypocalcemia secondary to hypoparathyroidism. Am J Med Sci.

[17] Catalano A, Basile G, Lasco A (2012). Hypocalcemia: a sometimes overlooked cause of heart failure in the elderly. Aging Clin Exp Res.

[18] Avsar A, Dogan A, Tavli T (2004). A rare cause of reversible dilated cardiomyopathy: hypocalcemia. Echocardiography.

